# Patient-reported outcomes in low anterior resection syndrome: a comparison of open and robotic surgery

**DOI:** 10.1007/s00384-026-05194-8

**Published:** 2026-07-17

**Authors:** Elisabeth Ammer, Sophie Winter, Christoph Ammer-Herrmenau, Johannes Moritz Riebeling, Philipp Horvath, Thomas Asendorf, Michael Ghadimi, Marian Grade

**Affiliations:** 1 Department of General, Visceral and Pediatric Surgery, University Medical Center Göttingen, Göttingen, Germany; 2 Department of Gastroenterology, Gastrointestinal Oncology and Endocrinology, University Medical Center Göttingen, Göttingen, Germany; 3 Department of Medical Statistics, University Medical Center Göttingen, Göttingen, Germany

**Keywords:** Rectal cancer, Robotic surgery, Low anterior resection syndrome, LARS score, PROM

## Abstract

**Purpose:**

Despite recent advances in multimodal treatment strategies, radical tumor resection remains a cornerstone in the management of patients with rectal cancer. However, a substantial proportion of patients develop postoperative functional impairment known as low anterior resection syndrome (LARS). Emerging evidence suggests that robotic-assisted surgery may offer superior functional outcomes compared to other approaches. Therefore, this study aimed to compare the incidence and severity of LARS after robotic and open surgery using a patient-reported outcome measure (PROM).

**Methods:**

This retrospective, single-center cohort study included patients with primary adenocarcinoma of the sigmoid colon or rectum who underwent open or robotic oncological resection between 2014 and 2024. Eligible participants completed a standardized, validated questionnaire to assess their individual LARS score. The LARS scores were subsequently correlated with selected clinicopathological characteristics.

**Results:**

LARS questionnaires were analyzed in 184 patients. The incidence of major LARS was comparable between open and robotic surgery (39.1% vs. 39.2%), as was the rate of minor LARS (20% vs. 15%), with no statistically significant difference observed in multivariable ordinal regression (*p* = 0.451). Independent risk factors for LARS included neoadjuvant treatment, hand-sewn coloanal anastomosis, formation of a protective stoma, tumor location in the middle and lower rectum, and total mesorectal excision.

**Conclusion:**

LARS remains a significant clinical challenge, irrespective of the surgical approach. This PROM-based finding is particularly relevant for preoperative counseling and shared decision-making, as it suggests that open surgery is not inherently associated with worse functional outcomes in terms of LARS. Future research should aim to identify the biological, anatomical, and clinical determinants underlying the variable susceptibility to LARS.

**Supplementary Information:**

The online version contains supplementary material available at 10.1007/s00384-026-05194-8.

## Introduction

Despite recent advances in multimodal treatment concepts, radical tumor resection with oncological lymphadenectomy remains a cornerstone in the management of patients with rectal cancer [1, 2]. Although current evidence indicates comparable oncological outcomes between minimally invasive techniques and open surgery, robotic surgery has gained increasing popularity [3–9]. This is largely attributed to its ability to overcome many technical limitations of conventional laparoscopy, offering enhanced ergonomics and precision. Furthermore, it has been hypothesized that robotic surgery may lead to superior functional outcomes compared to other surgical approaches, although this remains to be fully substantiated [3–9].

In this context, a considerable proportion of patients undergoing radical surgical resection develop a postoperative functional disorder known as low anterior resection syndrome (LARS). This multifactorial and complex condition is characterized by symptoms such as impaired bowel continence, defecatory urgency, and increased stool frequency [10–19]. To enable objective assessment of individual LARS severity, a validated, questionnaire-based scoring system was developed over a decade ago and has since become the established standard for evaluating bowel function following rectal cancer surgery [20–25].

To the best of our knowledge, the incidence and severity of LARS have not yet been compared between open and robotic surgery in a standardized, patient-reported outcome measure (PROM)–based setting. Therefore, this study aimed to investigate whether the implementation of robotic surgery is associated with lower rates of LARS compared to open surgery.

## Patients and methods

### Study design and eligibility criteria

Patients with primary adenocarcinoma of the sigmoid colon or rectum were identified from the institutional cancer database and clinical information system. Given that oncological resection of sigmoid colon cancers at our institution involves resection of the upper rectum and partial mesorectal excision (PME), these patients were included in the study. All participants underwent either open or robotic radical surgical resection at the University Medical Center Göttingen, a tertiary referral center in Germany, between February 2014 and May 2024. Robotic surgery was performed using the da Vinci® Si Surgical System (up to August 2017) or the da Vinci® Xi Surgical System (from September 2017). Patients were not randomized to surgical approach; instead, the choice of procedure was determined by clinical factors (e.g., tumor location, patient comorbidities), surgeon expertise, and patient preference. With increasing experience and proficiency in robotic surgery, the proportion of patients undergoing robotic resection gradually increased over time.

Eligible participants who were alive at the time of LARS assessment and fluent in German received the LARS questionnaire via postal mail, accompanied by a prepaid, self-addressed envelope.

Exclusion criteria were defined as follows:

- Conventional laparoscopic surgery;
- Absence of gastrointestinal continuity at the time of assessment (e.g., permanent colostomy);
- Time interval from primary tumor resection or temporary stoma reversal to LARS assessment less than 6 months;
- Local tumor recurrence;
- Need for pelvic reoperation (e.g., secondary coloanal anastomosis due to anastomotic leakage or surgery for local recurrence);
- Patients already referred for revision surgery due to severe (major) LARS;
- Prior colectomy;
- Familial adenomatous polyposis (FAP);
- Other conditions that precluded successful completion of the questionnaire (e.g., stroke, dementia, and schizophrenia).

Patients with distant metastases were included if they had an adequate performance status and manageable disease burden.

### Outcomes

The LARS questionnaire, a validated PROM, comprises a 5-item scoring system assessing postoperative bowel function [23–25]. Individual item scores are summed to yield a total LARS score ranging from 0 to 42. Based on established cut-off values, the total score is classified into three categories [21–25]: no LARS (0–20 points), minor LARS (21–29 points), and major LARS (30–42 points). The validated German version of the LARS questionnaire was used [23], with only minor adjustments to the graphical layout to align with the institutional corporate design.

In addition, a supplementary question was added to assess health-related quality of life (QoL), as previously described in the literature [21, 25]. This item evaluates the extent to which bowel function impacts the patient’s daily life, using the following response categories: “not at all,” “very little,” “somewhat,” and “a lot” (see Supplementary Figure 1).

The primary endpoint was the strength of association between surgical approach (open versus robotic surgery) and the presence of LARS. Secondary endpoints included a post hoc multivariable analysis to identify clinicopathological risk factors associated with the development of LARS and an evaluation of the association between LARS severity and patient-reported quality of life.

### Clinical data

Data were extracted from the institutional cancer database and clinical information system. Demographic characteristics included age, sex, body mass index (BMI), and American Society of Anesthesiologists (ASA) classification. Tumor-related variables comprised primary tumor stage, nodal status, and resection margin status. Information on neoadjuvant therapy was recorded. Surgical details included the type of procedure (open vs. robotic surgery), the da Vinci® surgical system used (Si or Xi), conversion rate, type of mesorectal excision (total mesorectal excision (TME) or partial mesorectal excision (PME)), anastomotic technique (hand-sewn vs. stapled), and stoma formation. Postoperative outcomes included anastomotic leakage. Additionally, the time intervals from primary tumor resection and, if applicable, from temporary stoma reversal to LARS assessment were documented.

### Ethics

This study was approved by the Ethics Committee of the University Medical Center Göttingen (application number 18/5/24). Informed consent was obtained from all participants prior to inclusion.

### Statistical analysis

Data analysis was performed using R (v 4.4 or greater) and R-Studio. Continuous variables are presented as mean ± standard deviation (SD) and categorical variables as absolute and relative frequencies. A total of 12 potential confounders were evaluated: ten discrete and two continuous variables. To avoid overfitting, a preselection of potential confounding variables was conducted using an univariable regression with these variables and the endpoint variable (surgical approach for the primary endpoint and LARS for the secondary endpoint). All variables with a *p* value < 0.05 were considered to potentially influence the analyses. For nonbinary ordinal variables, an univariable and multivariable ordinal regression was performed including the predefined confounders as covariates. Pairwise comparisons were conducted using the emmeans package (version 1.11.1). Multiple testing correction was applied using the Benjamini–Hochberg procedure to control the false discovery rate. A two-sided *p* value < 0.05 was considered statistically significant.

## Results

**Fig. 1:**
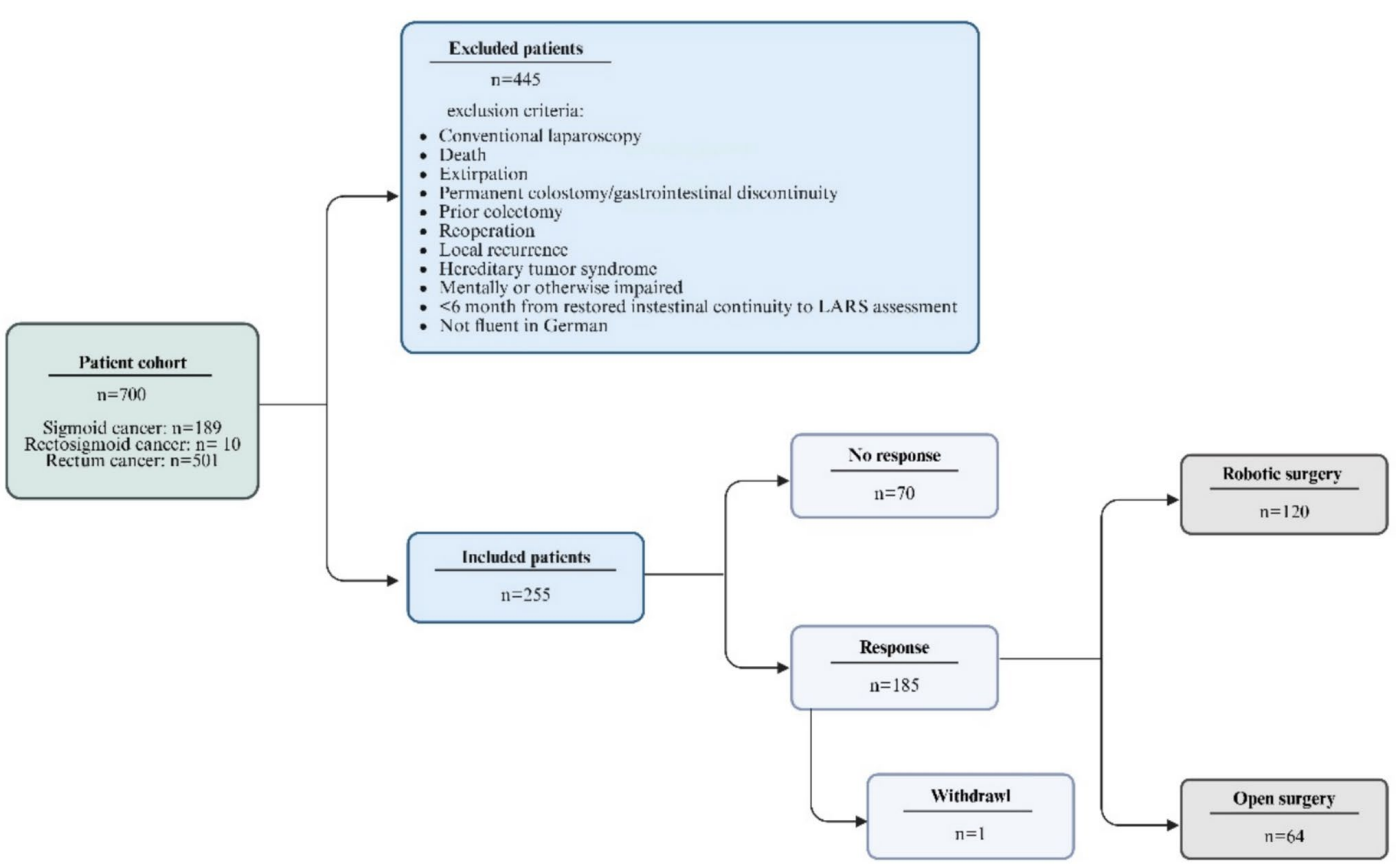
Strengthening the reporting of observational studies in epidemiology (STROBE) flowchart for selection of patients

A total of 700 patients with rectal or sigmoid colon cancer underwent radical surgical resection between February 2014 and May 2024 at the University Medical Center Göttingen (Fig. 1). Of these, 255 patients met the predefined inclusion criteria and were invited to complete the LARS questionnaire. A total of 185 completed questionnaires were returned, representing a response rate of 72.5%. After one participant withdrew consent, the final cohort comprised 184 patients: 120 patients in the robotic surgery group and 64 patients in the open surgery group.

### Patient baseline characteristics, surgical details, and histopathological outcomes

Baseline characteristics, surgical procedures, and histopathological outcomes are summarized in Table 1. The final cohort consisted of 70 female and 114 male patients, with a mean age of 63 years (SD 10) and a mean BMI of 27 kg/m^2^ (SD 5). Both age and BMI were analyzed as continuous variables. A total of 67 patients (36.4%) received neoadjuvant treatment, primarily long-term chemoradiotherapy or total neoadjuvant treatment (TNT). Robotic surgery was the predominant approach, performed in 65% of cases (120/184), with the da Vinci® Xi system used in the majority of procedures. TME was performed in 88 patients (47.8%), while PME was performed in 96 patients (52.2%). A protective stoma was created in 90 patients (48.9%). The mean time from primary tumor resection to LARS assessment was 56 months (SD 33), and the mean time from temporary stoma reversal to LARS assessment was 57 months (SD 32).

**Table 1:** Baseline characteristics, surgical details, and histopathological outcomes

Clinical variable	Data
Gender, ***n*** (%)	
Female	70 (38%)
Male	114 (62%)
Mean BMI (SD)	27 (5)
ASA classification, ***n*** (%)	
1–2	141 (77%)
3–4	43 (23%)
Neoadjuvant treatment, ***n*** (%)	67 (36%)
Short-course radiotherapy	2
Long-term chemoradiotherapy	45
Total neoadjuvant therapy	18
Chemotherapy	2
Mean age at tumor resection, years (SD)	63 (10)
Surgical approach, ***n*** (%)	
Open surgery	64 (35%)
Robotic surgery	120 (65%)
Conversion, ***n*** (%)	5 (4.2%)
Tumor localization, ***n*** (%)	
Sigmoid colon	61 (33%)
Upper rectum	40 (22%)
Middle rectum	57 (31%)
Lower rectum	26 (14%)
Type of mesorectal excision, ***n*** (%)	
Partial mesorectal excision	96 (52%)
Total mesorectal excision	88 (48%)
Anastomosis	
Anastomotic technique, ***n*** (%)	
Hand-sewn coloanal anastomosis	11 (6%)
Stapler	173 (94%)
Intraoperative anastomotic tightness, ***n*** (%)	
Leakage	7 (3.8%)
No leakage	177 (96%)
Anastomotic insufficiency, ***n*** (%)	10 (5.4%)
Da Vinci system, ***n*** (%)	
Xi	104
Si	16
Formation of a protective stoma, ***n*** (%)	90 (48.9%)
Histopathology (surgery after neoadjuvant therapy), ***n*** (%)	67 (36%)
T stage	
ypT0	17
ypT1	5
ypT2	25
ypT3	19
ypT4	1
N stage	
ypN0	59
ypN1	7
ypN2	1
Histopathology (primary surgery, no neoadjuvant therapy), ***n*** (%)	117 (64%)
T stage	
pT1	30
pT2	37
pT3	41
pT4	9
N stage	
pN0	78
pN1	28
pN2	11
Resection margin status, ***n*** (%)	
R0	184 (100%)
Mean time from tumor resection to LARS assessment, months (SD)	56 (33)
Mean time from temporary stoma reversal to LARS assessment, months (SD)	57 (32)

*BMI* body mass index, *ASA* American Society of Anesthesiologists

### LARS incidence and severity in open vs. robotic surgery

**Fig. 2:**
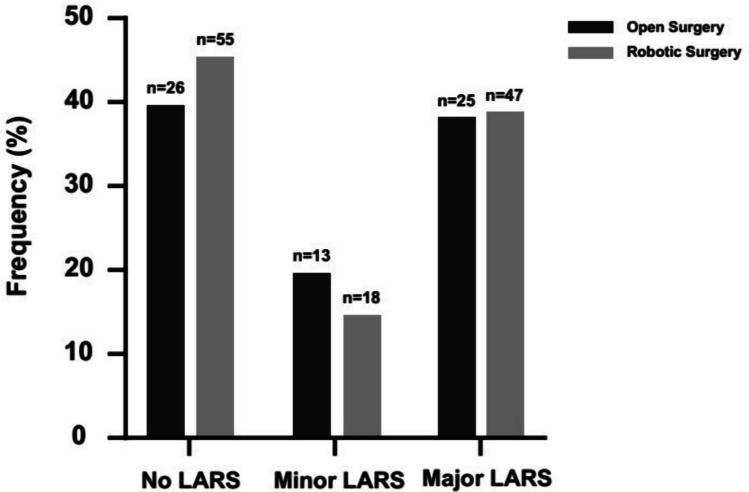
Incidence and severity of LARS for both open surgery and robotic surgery

Overall, the incidence of minor LARS was 17% (*n* = 31) and that of major LARS was 39% (*n* = 72), while 81 patients (44%) exhibited no symptoms of LARS (Table 2). When comparing surgical approaches, the overall LARS rate was 59.4% (38 of 64 patients) in the open surgery group and 54.2% (65 of 120 patients) in the robotic surgery group (Fig. 2). Specifically, minor LARS was observed in 20.3% (*n* = 13) of patients in the open surgery group and 15.0% (*n* = 18) in the robotic surgery group. Major LARS occurred in 39.1% (*n* = 25) after open surgery and 39.2% (*n* = 47) after robotic surgery. In univariable ordinal regression analysis, no significant association was found between surgical approach and LARS severity (*p* = 0.708).

**Table 2:** Comparison of surgical procedures in terms of the development of LARS

Variable	Open surgery (*n* = 64)	Robotic surgery (*n* = 120)	Total (*n* = 184)
LARS, ***n*** (%)			
No LARS	26 (40.6%)	55 (45.8%)	81 (44.0%)
All LARS	38 (59.4%)	65 (54.2%)	103 (56.0%)
Minor LARS	13 (20.3%)	18 (15.0%)	31 (16.9%)
Major LARS	25 (39.1%)	47 (39.2%)	72 (39.1%)

To identify potential confounders, we conducted univariable logistic regression to assess differences in baseline characteristics between the open and robotic surgery. As shown in Table 3, gender, ASA classification, and age at tumor resection were significantly different between groups and were therefore included as covariates in the multivariable ordinal regression model. Multivariable analysis confirmed the absence of a significant association between surgical approach and LARS severity (adjusted *p* = 0.451; odds ratio [OR] 0.79), as illustrated in Table 4. Furthermore, none of the other evaluated variables showed a statistically significant association with LARS severity.

**Table 3:** Univariable logistic regression between open and robotic surgery to identify potential confounding variables

Clinical variable	Open surgery	Robotic surgery	*p* value	OR	95% CI
Gender, ***n***			**0.009**	**2.45**	1.28–4.90
Female	16	54			
Male	48	66			
Mean BMI (SD)	28 (5)	27 (4)	0.091	0.95	0.89–1.01
ASA classification, ***n***			**< 0.001**	**0.21**	0.10–0.43
1–2	37	104			
3–4	27	16			
Mean age at tumor resection, years (SD)	66 (9)	61 (10)	**0.005**	**0.95**	0.92–0.98
Tumor localization, ***n***			0.6		
Lower rectum	9	17		0.99	0.38–2.68
Middle rectum	17	40		1.24	0.57–2.70
Upper rectum	17	23		0.71	0.31–1.62
Sigmoid colon	21	40			
Neoadjuvant therapy, ***n***	23	44	> 0.9	1.03	0.55–1.96
Anastomotic technique, ***n***			0.4	0.62	0.18–2.23
Hand-sewn anastomosis	5	6			
Stapled anastomosis	59	114			
Intraoperative anastomotic tightness, ***n***			0.6	0.7	0.15–3.65
Leakage	3	4			
No leakage	61	116			
Formation of a protective stoma, ***n***	32	58	0.8	0.94	0.51–1.72
Type of mesorectal excision, ***n***			0.9	0.94	0.51–1.73
Partial mesorectal excision	34	62			
Total mesorectal excision	30	58			
Postoperative anastomotic leak, ***n***	5	5	0.3	0.51	0.14–1.91

*OR* odds ratio, *CI* confidence interval, *BMI* body mass index, *ASA* American Society of Anesthesiologists, values in bold indicate results that are statistically significant at the 0.05 significance level.

**Table 4:** Multivariable ordinal regression for confounding variables and surgical approach regarding the development of LARS

	OR	95% CI	*p* value
Surgical approach	0.79	0.17–1.41	0.451
Age at tumor resection	0.98	0.95–1.02	0.313
ASA classification	1.20	0.53–1.88	0.592
Gender	1.79	1.21–2.37	0.05

*OR* odds ratio, *CI* confidence interval, *ASA* American Society of Anesthesiologists

Given the previously reported higher incidence of LARS following TME compared to PME [20, 24], we conducted a subgroup analysis restricted to patients who underwent TME. In this cohort, LARS was observed in 81% of patients, comprising 16% with minor LARS and 65% with major LARS (Table 5).

**Table 5:** Comparison of surgical procedures in terms of the development of LARS restricted to patients who underwent TME

Variable	Open surgery (*n* = 30)	Robotic surgery (*n* = 58)	Total (*n* = 88)
LARS, ***n*** (%)			
No LARS	7 (23.3%)	10 (17.2%)	17 (19.3%)
All LARS	23 (76.7%)	48 (82.8%)	71 (80.7%)
Minor LARS	6 (20.0%)	8 (13.8%)	14 (15.9%)
Major LARS	17 (56.7%)	40 (69.0%)	57 (64.8%)

When stratified by surgical approach, the overall incidence of LARS was 77% (23 of 30 patients) in the open surgery group and 83% (48 of 58 patients) in the robotic surgery group. Among patients undergoing open surgery, 20% (6 of 30 patients) experienced minor LARS and 57% (17 of 30 patients) experienced major LARS. Correspondingly, in the robotic surgery group, 14% (8 of 58 patients) developed minor LARS and 69% (40 of 58 patients) developed major LARS.

A comprehensive assessment of potential confounding variables revealed no significant associations with LARS occurrence (Supplementary Table 1). Furthermore, univariable ordinal regression analysis showed no significant association between surgical approach and LARS severity LARS among patients undergoing TME (OR 1.63, 95% CI 0.67–3.96; *p* = 0.30).

LARS symptoms are generally expected to stabilize within 12 to 24 months after surgery [26, 27]. Given the variability in the time interval between tumor resection (or stoma reversal, where applicable) and LARS assessment, we conducted a subgroup analysis stratifying patients according to the duration between restoration of bowel continuity and questionnaire completion (< 24 months vs. ≥ 24 months). Consistent with our primary findings, no significant difference in LARS prevalence was observed between surgical approaches in either subgroup (Table 6).

**Table 6:** Multivariable ordinal regression analysis assessing the association between the time interval from restoration of bowel continuity to questionnaire completion, surgical approach, and the development of LARS

	OR	95% CI	*p* value
Surgical approach	0.94	0.37–1.52	0.85
Time from restoration of continuity to questionnaire	1.77	1.11–2.43	0.09

*OR* odds ratio, *CI* confidence interval

### Clinical risk factors for the development of LARS

Following the observation that robotic surgery was associated with LARS rates comparable to those of open surgery, we conducted a post hoc multivariable analysis to identify clinical factors independently associated with the development and severity of LARS. After correction for multiple testing, five clinical variables remained statistically significant (Table 7):

- Neoadjuvant therapy (adjusted *p* < 0.001);
- Anastomotic technique (adjusted *p* = 0.019);
- Formation of a protective stoma (adjusted *p* < 0.001);
- Tumor location (sigmoid colon vs. middle rectum: adjusted *p* < 0.001; sigmoid colon vs. lower rectum: adjusted *p* < 0.001);
- Type of mesorectal excision (adjusted *p* < 0.001).

**Table 7:** Post hoc analysis of clinical risk factors for the development of LARS. Univariable ordinal regressions were performed with a false discovery rate as multiple test correction (adjusted *p* value)

Clinical variable	No LARS	Minor LARS	Major LARS	*p* value	OR	95% CI	Adj. *p* value
Gender, ***n***				0.057	1.73	0.99–3.04	0.11
Female	25	12	33				
Male	56	19	39				
Mean BMI (SD)	27 (5)	27 (5)	27 (5)	0.9	1.0	0.94–1.05	0.9
ASA classification, ***n***				0.8	1.08	0.58–2.01	0.9
1–2	65	19	57				
3–4	16	12	15				
Mean age at tumor resection, years (SD)	63 (10)	66 (7)	61 (9)	0.3	0.99	0.96–1.01	0.5
Conversion, ***n***	2	0	3	0.5	1.82	0.29–14.3	0.7
Neoadjuvant therapy, ***n***	11	7	49	**< 0.001**	**9.72**	5.08–19.4	**< 0.001**
Anastomotic technique, ***n***				**0.008**	**16.9**	3.12–314	**0.019**
Hand-sewn anastomosis	1	0	10				
Stapler	80	31	62				
Intraoperative anastomotic tightness, ***n***				0.8	0.84	0.20–3.32	0.9
Leakage	3	2	2				
No leakage	78	29	70				
Protective stoma, ***n***	17	14	59	**< 0.001**	**10.3**	5.57–19.7	**< 0.001**
Type of mesorectal excision, ***n***				<** 0.001**	**0.11**	0.06–0.20	**< 0.001**
Partial mesorectal excision	64	17	15				
Total mesorectal excision	17	14	57				
Anastomotic leak, ***n***	3	1	6	0.2	2.25	0.65–8.93	0.4
Tumor localization, ***n***				**< 0.001**			** < 0.001**
Sigmoid colon	45	9	7	-	-		-
Upper rectum	21	10	9	0.033	2.42	1.08–5.53	0.071
Middle rectum	12	10	35	< 0.001	11.4	5.28–25.8	**< 0.001**
Lower rectum	3	2	21	< 0.001	29.6	10.0–103	**< 0.001**

*OR* odds ratio, *BMI* body mass index, *CI* confidence interval, *ASA* American Society of Anesthesiologists,  values in bold indicate results that are statistically significant at the 0.05 significance level.

These findings indicate that the use of neoadjuvant therapy, a hand-sewn coloanal anastomosis, creation of a protective stoma, tumor location in the middle or lower rectum, and TME are independent predictors of increased LARS severity, regardless of the surgical approach employed.

### LARS and quality of life

We further investigated the association between LARS severity and health-related quality of life. A majority of patients reported that their bowel function significantly impacts their daily lives: 19.0% reported “not at all,” 21.2% reported “very little,” 33.2% reported “somewhat,” and 26.6% reported “a lot” (Table 8).

**Table 8:** Impact of LARS on quality of life

Impact on quality of life, *n* (%)	No LARS (*n* = 81)	Minor LARS (*n* = 31)	Major LARS (*n* = 72)	Total (*n* = 184)
“Not at all”	35 (43.2%)	0 (0%)	0 (0%)	35 (19.0%)
“Very little”	29 (35.8%)	7 (22.6%)	3 (4.2%)	39 (21.2%)
“Somewhat”	15 (18.5%)	19 (61.3%)	27 (37.5%)	61 (33.2%)
“A lot”	2 (2.5%)	5 (16.1%)	42 (58.3%)	49 (26.6%)

**Fig. 3:**
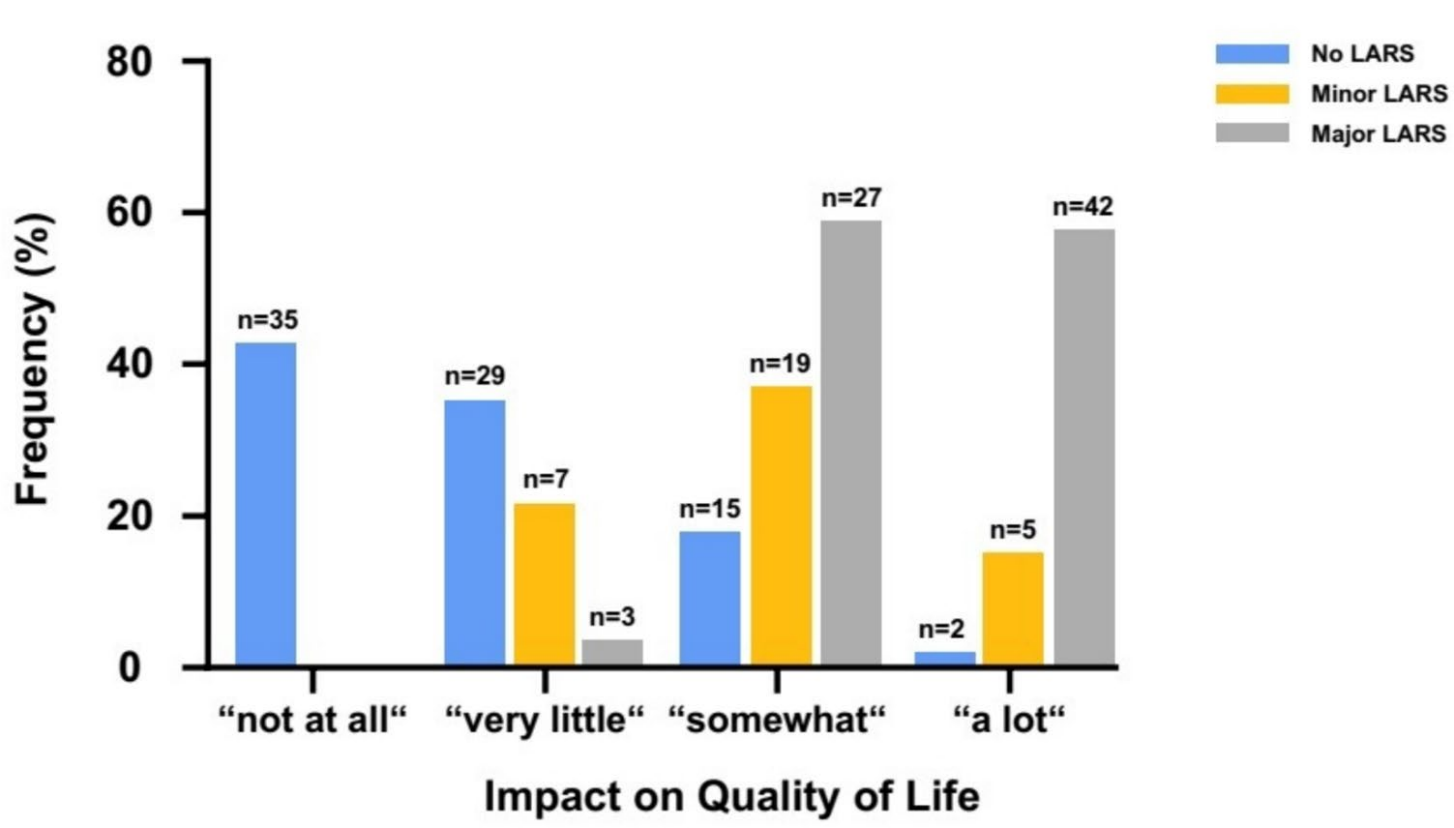
Correlation between LARS and its impact on quality of life

As shown in Fig. 3, patients without LARS experienced minimal impairment in daily functioning, with 79.0% reporting high QoL ratings. In stark contrast, 95.8% of patients with major LARS reported poor QoL, and no patient in this group reported no impact on their quality of life (Fig. 3, Table 8). Univariable ordinal regression analysis revealed a highly significant association between LARS severity and QoL (no LARS vs. minor LARS: *p* < 0.001, OR = 14.0; no LARS vs. major LARS: *p* < 0.001, OR = 98.0; minor LARS vs. major LARS: *p* < 0.001, OR = 6.5).

To account for potential confounding factors, we preselected variables that differed significantly across LARS severity groups. As shown in Table 7, neoadjuvant therapy, anastomotic technique, formation of a protective stoma, tumor localization, and type of mesorectal excision were significantly associated with LARS severity and were therefore included in the multivariable ordinal regression model (Table 9). After adjustment for these confounders, the association between LARS severity and QoL remained highly significant (Table 10):

- No LARS vs. minor LARS: *p* < 0.001, OR = 12.92;
- No LARS vs. major LARS: *p* < 0.001, OR = 49.84;
- Minor LARS vs. major LARS: *p* = 0.016, OR = 3.86.

**Table 9:** Multivariable ordinal regression for quality of life

	OR	95% CI	*p* value
No LARS–minor LARS	12.92	11.99–13.84	< 0.001
No LARS–major LARS	49.84	48.82–50.85	< 0.001
Neoadjuvant therapy	0.55	0.43–1.54	0.24
Anastomotic technique	1.82	0.11–3.53	0.493
Formation of a protective stoma	3.67	2.16–5.18	0.092
Type of mesorectal excision	2.00	0.34–3.66	0.412
Tumor localization			
Sigmoid colon–upper rectum	1.40	0.56–2.24	0.436
Sigmoid colon–middle rectum	4.67	3.32–6.02	0.025
Sigmoid colon–lower rectum	8.03	6.35–9.71	0.0015

*OR* odds ratio, *CI* confidence interval

**Table 10:** Pairwise post hoc analysis of multivariable ordinal regression for the impact of LARS on quality of life

	OR	95% CI	*p* value
No LARS–minor LARS	12.92	0.85–1.00	< 0.001
No LARS–major LARS	49.84	1.0–1.04	< 0.001
Minor LARS–major LARS	3.86	0.70–1.22	0.016

*OR* odds ratio, *CI* confidence interval

These findings underscore that LARS severity is an independent predictor of impaired quality of life, even after adjusting for key clinical and surgical factors.

## Discussion

Radical tumor resection remains a cornerstone in the management of patients with rectal cancer. In recent years, robotic rectal surgery has gained increasing popularity and is expected to surpass laparoscopic surgery in the near future [6, 28]. Despite this trend, the advantages of robotic surgery remain a subject of ongoing debate.

In this study, we hypothesized that robotic surgery would be associated with lower rates of LARS compared to open surgery. However, we observed no significant difference in LARS incidence between the two approaches: 59.4% in the open surgery group versus 54.2% in the robotic surgery group (*p* = 0.451). This finding was unexpected, given the theoretical advantages of robotic surgery, such as enhanced visualization, improved dexterity, and greater precision, features that were anticipated to translate into better functional outcomes. One possible explanation lies in the pathophysiology of LARS itself. Emerging evidence suggests that postoperative alterations in distal colonic motility, resulting from dissection of the sigmoid colon or rectum, play a central role in the development of LARS [29, 30]. These intrinsic changes may outweigh the technical benefits of robotic surgery, at least with respect to bowel function.

The question of whether robotic surgery offers advantages over open surgery in other functional domains, such as sexual or bladder function, remains unresolved and warrants further investigation. Moreover, from a broader clinical perspective, the underlying factors contributing to the substantial interindividual variability in LARS development remain poorly understood, regardless of surgical approach. While approximately 15% of individuals in the general population report symptoms consistent with major LARS, a substantial proportion of patients undergoing rectal cancer surgery do not develop such symptoms, highlighting the complex interplay of biological, surgical, and individual factors [31, 32].

To the best of our knowledge, this is the first study to directly compare LARS incidence and severity between open and robotic surgery using a validated, patient-reported outcome measure. Yet prior studies have compared robotic surgery with laparoscopic surgery. Zhang et al. conducted a retrospective, propensity score-matched analysis of 342 patients and reported a lower incidence of LARS in the robotic group (56.4% vs. 76.4%) over an 18-month follow-up period [33]. The overall incidence of LARS was 68.7%, with minor and major LARS rates of 32.7% and 36.0%, respectively. In contrast, Bolton et al. found no significant difference in LARS rates between robotic and laparoscopic surgery in the ROLARR trial (75.8% vs. 88.9%; *p* = 0.694), with an overall LARS incidence of 82.6% (minor LARS: 19.7%; major LARS: 62.9%) in 132 patients [34].

More recently, Feng et al. reported superior oncological and functional outcomes in a large randomized clinical trial comparing robotic and laparoscopic surgery (*n* = 1171) [9]. After 3 years of follow-up, the robotic group demonstrated lower locoregional recurrence (1.5% vs. 4.0%; *p* = 0.03) and improved disease-free survival (87.2% vs. 83.4%; *p* = 0.04). Additionally, patients in the robotic group reported better bladder and sexual function following robotic surgery and fewer defecation problems in the first 6 months postoperatively. However, bowel function was assessed using the Wexner Continence Grading Scale rather than the LARS questionnaire, limiting direct comparability with our findings.

According to the literature, the overall incidence of LARS ranges from 40 to 60% for major LARS, with up to 90% of patients affected when minor cases are included [15, 21, 26, 27, 31, 34, 35]. In our cohort, the rates of minor LARS (17%) and major LARS (39%) were lower than reported in many previous studies. This may be attributed to the relatively high proportion of partial mesorectal excisions (52%) in our cohort compared to total mesorectal excisions (48%). However, subgroup analysis restricted to patients who underwent TME revealed no significant difference in LARS incidence between open and robotic surgery. Furthermore, no significant differences were observed in the distribution of TME and PME between the open and robotic surgery groups. This suggests that the extent of mesorectal excision did not confound the comparison between surgical approaches.

We further confirmed a strong association between LARS severity and impaired quality of life. Patients with major LARS reported significantly worse health-related QoL, consistent with findings from large-scale studies demonstrating reduced physical, emotional, cognitive, and social functioning in this group [15, 25, 36, 37].

In a post hoc analysis, we identified several independent clinical risk factors for LARS: neoadjuvant therapy, hand-sewn coloanal anastomosis, formation of a protective stoma, tumor localization in the middle or lower rectum, and TME. These findings align with previous literature [15–17, 26, 27, 34], reinforcing the reproducibility and generalizability of our results although sample size in our cohort is small and reduces the effect. Neoadjuvant radiotherapy has been consistently linked to increased LARS severity due to its potential effects on pelvic nerves and sphincter function [17, 38]. Similarly, stapled anastomoses have been previously associated with lower LARS risk compared to hand-sewn coloanal anastomoses [14, 24]. However, the small number of patients with hand-sewn anastomoses in our cohort limits the statistical power of the analysis, and thus, this finding should be interpreted with caution. Finally, the identification of a protective stoma as a risk factor may reflect its strong association with TME, which is frequently followed by stoma creation [1, 39].

Despite the finding that robotic and open surgery show similar LARS rates, limitations of our study must be acknowledged. *First*, this is a nonrandomized, retrospective single-center study with a cross-sectional PROM assessment, which may introduce selection bias. *Second*, a formal power calculation was not performed; instead, the cohort comprised all eligible patients available at the time of analysis. *Third*, there was variability in the time interval between tumor resection (or stoma reversal, where applicable) and LARS assessment. Given that LARS symptoms are generally considered to stabilize within 12–24 months after surgery, we conducted a subgroup analysis stratifying patients according to the duration between restoration of bowel continuity and questionnaire completion (< 24 months vs. ≥ 24 months). The association between surgical approach and LARS development remained nonsignificant across both time intervals. *Fourth*, the robotic surgery group was larger (*n* = 120) than the open surgery group (*n* = 64), and the groups were imbalanced with respect to gender and ASA classification—men were more prevalent in the open surgery group, while patients with lower ASA scores were more common in the robotic group. These imbalances may influence outcomes, although we adjusted for these variables in multivariable models. It should be noted that group matching was not performed, as it would have substantially reduced the sample size, and potentially compromised the statistical power and robustness of the analysis. One possible explanation for the higher ASA scores in the open surgery group is that not all patients are eligible for minimally invasive surgery, particularly those with significant pulmonary or cardiac comorbidities. However, subgroup analyses restricted to patients with ASA scores of 1–2 and 3–4 revealed no significant differences in LARS incidence between open and robotic surgery (data not shown). *Fifth*, our cohort included a relatively high proportion of partial mesorectal excisions (52%). It remains unclear whether a higher proportion of total mesorectal excisions would have revealed significant differences between surgical approaches. However, in a subgroup analysis restricted to patients who underwent TME, surgical approach was not significantly associated with LARS severity. *Sixth*, quality of life was not assessed using validated instruments such as the EORTC QLQ-CR29 or QLQ-C30 questionnaires. Instead, we included a supplementary question to evaluate the impact of bowel function on daily life, based on a previously published approach [21, 23], which may constrain the interpretability and generalizability of our findings.

In summary, our PROM-based study confirms that LARS remains a highly prevalent and clinically significant complication after radical cancer surgery, with a substantial negative effect on patients’ quality of life. Importantly, our results suggest that robotic surgery is not inherently associated with lower LARS rates compared to open surgery. Although this finding should be interpreted with caution due to the study’s retrospective, single-center design, it carries important clinical implications for patient counseling. Future research should aim to identify the biological, anatomical, and clinical determinants underlying the variable susceptibility to LARS.

## Supplementary Information

Below is the link to the electronic supplementary material.

ESM 1 (DOCX 569 KB)

## Data Availability

Data supporting the findings of this study are not publicly available due to privacy and ethical concerns. They are available from the corresponding author upon reasonable request. All data are securely stored in a controlled-access data storage at the University Medical Center Göttingen, in compliance with data protection and ethical guidelines.
