# Supplementary material for: Patient-reported outcomes in low anterior resection syndrome: a comparison of open and robotic surgery
Source: Int J Colorectal Dis. 2026 Jul 17;41(1):122. doi: 10.1007/s00384-026-05194-8 (PMC13375658; doi:10.1007/s00384-026-05194-8)
Supplement: Supplementary file 1 — (DOCX 569 KB) [file 384_2026_5194_MOESM1_ESM.docx]

**Supplementary Data:**

| **Clinical variable** | **Open surgery** | **Robotic surgery** | **p-value** | **OR** | **95% CI** |
| --- | --- | --- | --- | --- | --- |
| **Gender, n** |  |  | 0.069 | 2.33 | 0.94-6.17 |
| Female | 9 | 29 |  |  |  |
| Male | 21 | 29 |  |  |  |
| **Mean BMI (SD)** | 27 (5) | 26 (4) | 0.2 | 0.94 | 0.85-1.03 |
| **ASA classification, n** |  |  | 0.3 | 0.53 | 0.17-1.66 |
| 1-2 | 23 | 50 |  |  |  |
| 3-4 | 7 | 8 |  |  |  |
| **Mean age at tumor resection, years (SD)** | 63 (9) | 62 (10) | 0.4 | 0.98 | 0.93-1.03 |
| **Tumor localization, n** |  |  | 0.8 |  |  |
| Lower rectum | 9 | 17 |  | 1.51 | 0.31-7.19 |
| Middle rectum | 17 | 36 |  | 1.69 | 0.38-7.21 |
| Upper rectum | 4 | 5 |  |  |  |
| Sigmoid colon | 0 | 0 |  |  |  |
| **Neoadjuvant therapy, n** | 23 | 40 | 0.4 | 0.68 | 0.23-1.81 |
| **Anastomotic technique, n** |  |  | 0.4 | 0.58 | 0.16-2.17 |
| Hand-sewn anastomosis | 5 | 6 |  |  |  |
| Stapled anastomosis | 25 | 52 |  |  |  |
| **Intraoperative anastomotic tightness, n** |  |  | 0.8 | 0.76 | 0.12-6.05 |
| Leakage | 2 | 3 |  |  |  |
| No leakage | 28 | 55 |  |  |  |
| **Formation of a protective stoma, n** | 29 | 56 | >0.9 | 0.97 | 0.04-10.5 |
| **Postoperative anastomotic leak, n** | 5 | 5 | 0.3 | 0.47 | 0.12-1.84 |
| OR: Odds Ratio  CI: Confidence Interval  BMI: Body Mass Index  ASA: American Society of Anesthesiologists | | | | |  |

**Table S1:** Univariable logistic regression between open and robotic surgery to identify potential confounding variables restricted to patients who underwent TME.


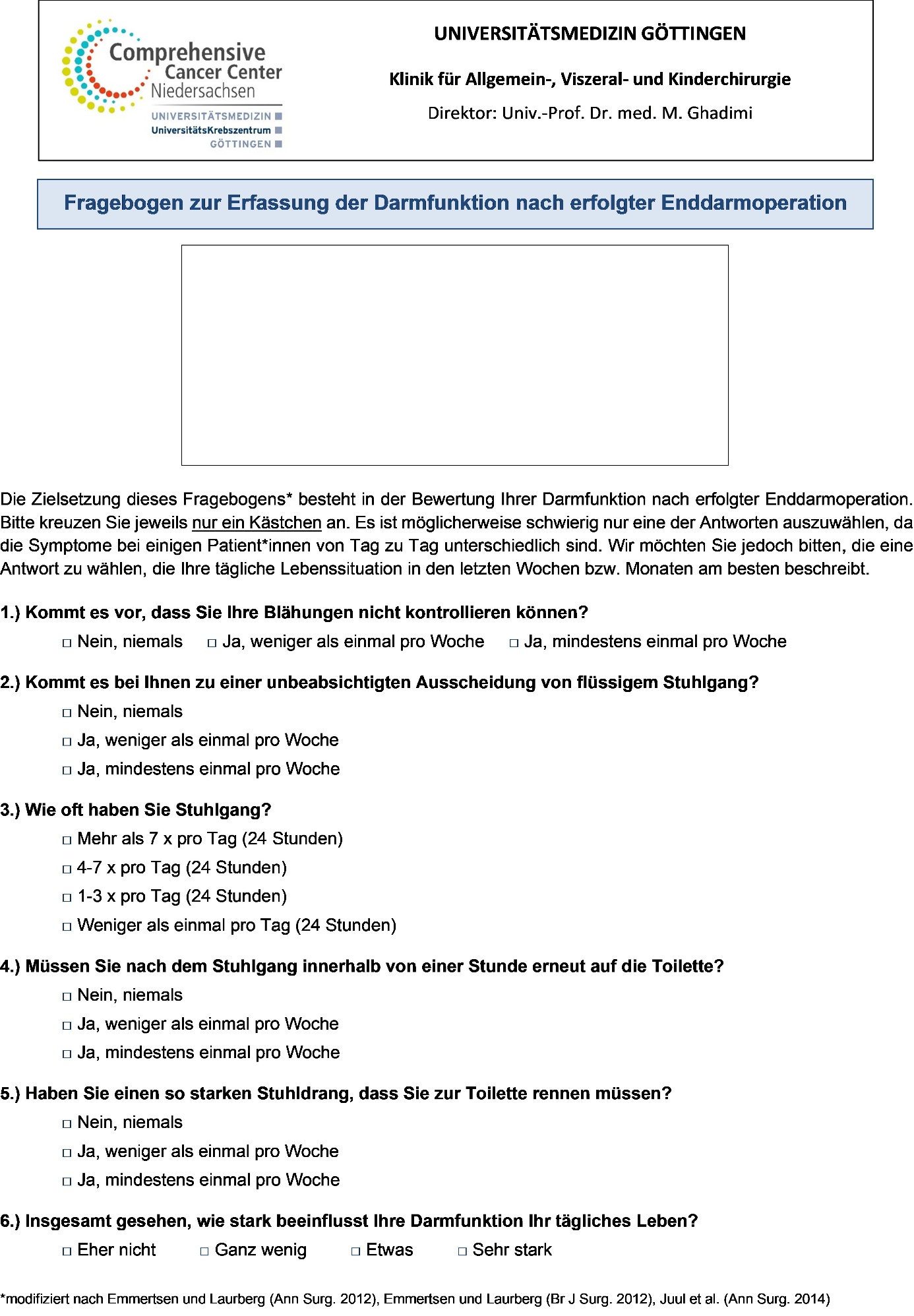


Figure S1: Validated German version of the LARS questionnaire.
